# Cognitive Resilience for the Prevention of Mild Cognitive Impairment in Subjective Cognitive Decline: A Monte Carlo Simulation of a Digital Therapeutic Targeting Dementia Risk Factors

**DOI:** 10.7759/cureus.83643

**Published:** 2025-05-07

**Authors:** Shaheen E Lakhan

**Affiliations:** 1 Biosciences, Global Neuroscience Initiative Foundation, Miami, USA; 2 Neurology, A.T. Still University School of Osteopathic Medicine in Arizona, Mesa, USA; 3 Neurology, Morehouse School of Medicine, Atlanta, USA; 4 Neurology, Western University of Health Sciences, Pomona, USA

**Keywords:** behavioral intervention, cognitive health, dementia prevention, digital therapeutics, mild cognitive impairment, modifiable risk factors, monte carlo simulation, neurodegenerative disease, personalized medicine, subjective cognitive decline

## Abstract

Introduction

Subjective cognitive decline (SCD) is an early marker of neurodegenerative disease and a target for preventative interventions. With advances in smartphone-based clinical interventions and understanding of modifiable risk factors for cognitive decline, this simulation study aimed to estimate the potential benefits of a prescription digital therapeutic (PDT) with multi-risk-factor modification on the cognitive trajectory of individuals with SCD. We constructed a Monte Carlo simulation to model progression to mild cognitive impairment (MCI) over a five-year period.

Methods

A virtual cohort of 10,000 patients with SCD was simulated over five years. Baseline annual risk of progression to MCI was set at 10%. A PDT was assumed to yield a 30% relative risk reduction, modulated by adherence levels (70% full, 20% partial, 10% none). Additionally, 14 modifiable dementia risk factors were modeled based on the 2024 Lancet Commission update, with each risk factor assigned a prevalence, modifiability flag, relative risk reduction, and effectiveness rate. Risk adjustments were applied multiplicatively, and outcomes were tracked annually.

Results

After five years, 6,487 (65%) of patients remained cognitively stable, 2,496 (25%) progressed to MCI, and 1,017 (10%) dropped out. These results represent a significant improvement over the expected 41% progression rate in untreated SCD populations. Reductions in physical inactivity, hypertension, hearing loss, and social isolation contributed substantially to outcome improvements, particularly when multiple risk factors were addressed concurrently.

Conclusion

The incorporation of a PDT with systematic modification of multiple modifiable dementia risk factors demonstrates a meaningful reduction in cognitive decline risk among individuals with SCD. These findings highlight the potential of integrated digital-first strategies to meaningfully delay cognitive decline and may inform future PDT trials and dementia prevention programs. Validation in prospective clinical trials is warranted to confirm these simulation-based findings.

## Introduction

Subjective cognitive decline (SCD) represents a self-perceived worsening of cognitive abilities without objective impairment on neuropsychological testing. SCD is now recognized as a high-risk condition for progression to mild cognitive impairment (MCI) and ultimately dementia, particularly Alzheimer’s disease [1]. While pharmacological treatment options remain limited for this preclinical stage, non-pharmacologic interventions, including lifestyle changes, behavioral coaching, and digital therapeutics, offer a promising avenue for early intervention.

Between 2017 and 2020, the Lancet Commission on Dementia Prevention, Intervention, and Care identified 12 modifiable risk factors that together account for approximately 40% of global dementia cases [2,3]. In its 2024 update, the Commission expanded this list to 14 risk factors, encompassing early- to late-life exposures, such as low education, hearing loss, hypertension, smoking, obesity, depression, physical inactivity, diabetes, excessive alcohol use, traumatic brain injury (TBI), air pollution, and social isolation [4]. These factors vary in their prevalence, impact, and degree of modifiability, making them ideal targets for personalized prevention strategies.

Prescription digital therapeutics (PDTs) are evidence-based digital interventions designed to prevent, manage, or treat medical conditions [5]. Regulated by the U.S. Food and Drug Administration (FDA), PDTs are delivered via smartphones and prescribed by licensed healthcare providers [6]. Unlike general wellness apps, PDTs undergo clinical validation through randomized controlled trials and are indicated for specific diagnoses or conditions, often as an alternative or adjunct to traditional pharmacotherapy. Their role in cognitive health is emerging, particularly for individuals at risk of neurodegenerative disease, where behavioral and lifestyle modification is critical but underutilized.

To evaluate the real-world potential of a multifaceted digital interventional approach, we developed a Monte Carlo simulation incorporating all PDT for cognitive resilience, addressing the Lancet-identified modifiable risk factors. The objective was to quantify the cumulative impact of these interventions on cognitive stability in individuals with SCD over a five-year period.

## Materials and methods

Simulation design

Monte Carlo simulation was chosen to capture probabilistic uncertainty in patient risk profiles and intervention response over time. This approach is widely used in health decision modeling and allows for simulation of realistic, heterogeneous population-level outcomes [7,8]. We conducted a Monte Carlo simulation using a synthetic population of 10,000 virtual individuals aged 60 and older with a diagnosis of SCD. Each patient was followed annually for five years. For each individual, we modeled the probability of progression to MCI, dropout from the intervention, and maintenance of cognitive stability. The simulation was developed in Python (version 3.11; Python Software Foundation, Wilmington, DE), along with NumPy (version 1.26) and SciPy (version 1.11) libraries for numerical computation and statistical modeling. The simulation was run for 10,000 iterations to ensure convergence and stable outcome distributions. Randomness was handled using a pseudo-random number generator with a fixed seed (NumPy RNG seed = 42) to ensure reproducibility. The model was fully probabilistic, with all parameters such as risk of disease progression, intervention adherence, dropout probability, and response to modifiable risk factors simulated as stochastic processes using predefined probabilities rather than fixed deterministic outcomes.

Intervention model

In this simulation, the PDT is modeled as a comprehensive, unified intervention designed to address the modifiable dementia risk factors identified by the 2024 Lancet Commission [4]. Rather than acting as an adjunct to individual lifestyle or clinical interventions, the PDT serves as the singular delivery system for multi-domain risk reduction. Features within the PDT include neuro-cognitive interventions, digital behavioral nudges, mood and sleep tracking, social engagement facilitation, remote monitoring and self-management for chronic conditions such as hypertension and diabetes, and education to promote healthy lifestyle changes [9].

The baseline annual risk of progression from SCD to MCI was set at 10%, consistent with previous longitudinal cohort studies [1,10]. The PDT was modeled to confer a 30% relative risk reduction (RRR) for disease progression. This assumption is based on evidence from randomized controlled trials and meta-analyses of multi-domain lifestyle interventions, such as the FINGER trial [11], and the findings of the Lancet Commission [2-4]. This effect was modulated by adherence: 70% of patients were classified as fully adherent (receiving full benefit), 20% as partially adherent (receiving 50% of the benefit), and 10% as non-adherent (no benefit). This model acknowledges the variability in patient engagement with the PDT, as highlighted in the literature [12], where adherence is a known moderator of effectiveness.

Risk factor assignment and modification

The values for prevalence, RRR, and effectiveness used in the simulation were derived from the 2024 Lancet Commission update [4] and supplemented by large epidemiological datasets and intervention studies as follows:

(1) Prevalence values were estimated based on age-adjusted frequencies in adults aged 60 and older, using data from the WHO [13], CDC [14], and peer-reviewed population studies [15,16]. For example, hearing loss prevalence in older adults is commonly cited as ~30%; hypertension affects ~40%; and physical inactivity affects ~50% in high-income settings.

(2) RRRs were drawn from the estimated population attributable fractions provided in the Lancet Commission [4]. For instance, hearing loss and low education were each estimated to contribute up to 7-8% of dementia risk, while smaller contributions were noted for factors such as obesity and alcohol use.

(3) Effectiveness values reflect a combination of empirical intervention success rates and feasibility estimates in real-world or digitally delivered settings. For example, PDTs may reduce physical inactivity in ~50% of patients [17], and hearing aid uptake when offered is around 50% [13]. These figures are conservative and intended to model average real-world effectiveness, not maximum possible efficacy.

Risk factors were modeled independently, and effects were applied multiplicatively. For example, hearing loss was assigned a prevalence of 30% in alignment with WHO [13] and CDC [14] epidemiological estimates for older adults, with an 8% RRR as reported in the Lancet Commission [4]. Intervention effectiveness of 50% reflects uptake of hearing aids in studies of digital hearing screening and outreach programs [18]. Hypertension (40% prevalence) was modeled with a 2% RRR based on midlife vascular studies, and a 70% effectiveness rate reflecting adherence to antihypertensive therapy in community-dwelling older adults [19]. Physical inactivity (50% prevalence, 3% RRR, 50% effectiveness) reflects sedentary behavior rates and the success of digital behavioral nudges in similar populations [20]. Smoking and alcohol use were assigned conservative RRRs (5% and 1%, respectively), consistent with their contributions to cerebrovascular and neurodegenerative risk in meta-analyses [21]. Social isolation was modeled with a 4% RRR and 60% effectiveness, supported by studies on digital interventions reducing loneliness [22]. These values reflect the intersection of behavioral, medical, and environmental modification feasibility.

In future iterations, we aim to model risk factor clustering (e.g., obesity and diabetes, depression and social isolation) using conditional probabilities and joint distributions to reflect the real-world collinearity among comorbid risk states. Although some clustering (e.g., metabolic syndrome components) is common, this simplifying assumption allowed for clearer attribution of individual and joint contributions. Future iterations may incorporate covariance among risk factors. Each patient was probabilistically assigned 0 or more of the 14 Lancet Commission [4] risk factors based on age-adjusted prevalence rates. For each risk factor, we applied an associated RRR and estimated an intervention effectiveness rate if the factor was modifiable (Table 1).

**Table 1:** Modeling Parameters for 14 Lancet Commission Dementia Risk Factors This table lists the 14 risk factors for dementia as defined by the 2024 Lancet Commission. Each factor is characterized by its estimated prevalence in older adults (aged ≥60), associated relative risk reduction (RRR) based on population-attributable fraction data, whether the factor is modifiable through a prescription digital therapeutic (PDT), and the assumed effectiveness of intervention in the Monte Carlo simulation model.

Risk Factor	Prevalence	RRR	Modifiable	Effectiveness
Low education	20%	7%	No	N/A
Hearing loss	30%	8%	Yes	50%
Hypertension	40%	2%	Yes	70%
Smoking	25%	5%	Yes	60%
Obesity	30%	1%	Yes	40%
Depression	20%	4%	Yes	50%
Physical inactivity	50%	3%	Yes	50%
Diabetes	25%	1%	Yes	50%
Excessive alcohol use	15%	1%	Yes	50%
Traumatic brain injury	10%	3%	No	N/A
Air pollution	30%	2%	Yes	30%
Social isolation	40%	4%	Yes	60%

If a patient had a modifiable risk factor, the annual progression risk was further reduced by a factor equal to (1 - RRR × effectiveness), applied multiplicatively. Total risk reductions were capped to avoid implausible outcomes, and annual dropout risk was fixed at 5% across all individuals.

Outcome measures

Three outcomes were recorded annually for each patient: remained cognitively stable, progressed to MCI, or dropped out. Once a patient progressed or dropped out, they were excluded from future simulation years. Summary statistics and cumulative outcome trajectories were compiled over the five-year horizon.

## Results

Among the 10,000 simulated patients with SCD, the cumulative outcomes after five years were as follows: 6,487 (65%) remained cognitively stable, 2,496 (25%) progressed to MCI, and 1,017 (10%) dropped out (Table 2). These results represent a significant improvement over the expected 41% progression rate in untreated SCD populations.

**Table 2:** Annualized Cognitive Outcomes Over Five Years in the Simulated SCD Cohort (n=10,000) with PDT This table summarizes the annual proportions of simulated patients with subjective cognitive decline (SCD) who remained cognitively stable, progressed to mild cognitive impairment (MCI), or dropped out of the intervention over a five-year period. The intervention included a prescription digital therapeutic (PDT) comprising risk factor modification. Percentages reflect cumulative outcomes for each category per year.

Year	Cognitively Stable	Progressed to MCI (Cumulative)	Dropped Out (Cumulative)
1	91%	5%	4%
2	84%	10%	6%
3	77%	16%	7%
4	71%	21%	8%
5	65%	25%	10%

**Figure 1:**
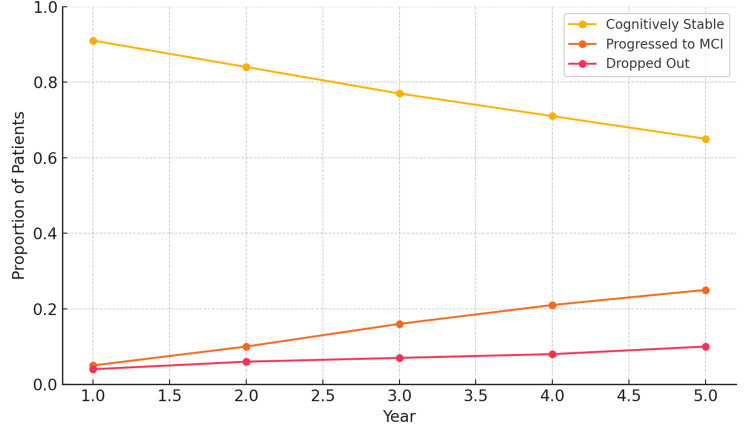
Cognitive Outcomes Over Five Years Among Subjective Cognitive Decline (SCD; n=10,000) Treated with Prescription Digital Therapeutic (PDT)

Year-by-year progression data revealed a steady decline in cognitive stability, with the most pronounced improvements observed in years three through five - suggesting a cumulative benefit over time (Figure 1). Notably, progression to MCI slowed more markedly in individuals with higher adherence to the digital therapeutic and multiple successfully modified risk factors. For example, patients with high PDT adherence and three or more modified risk factors had a five-year progression rate of just 18%, compared to 41% in those receiving no intervention.

**Figure 2:**
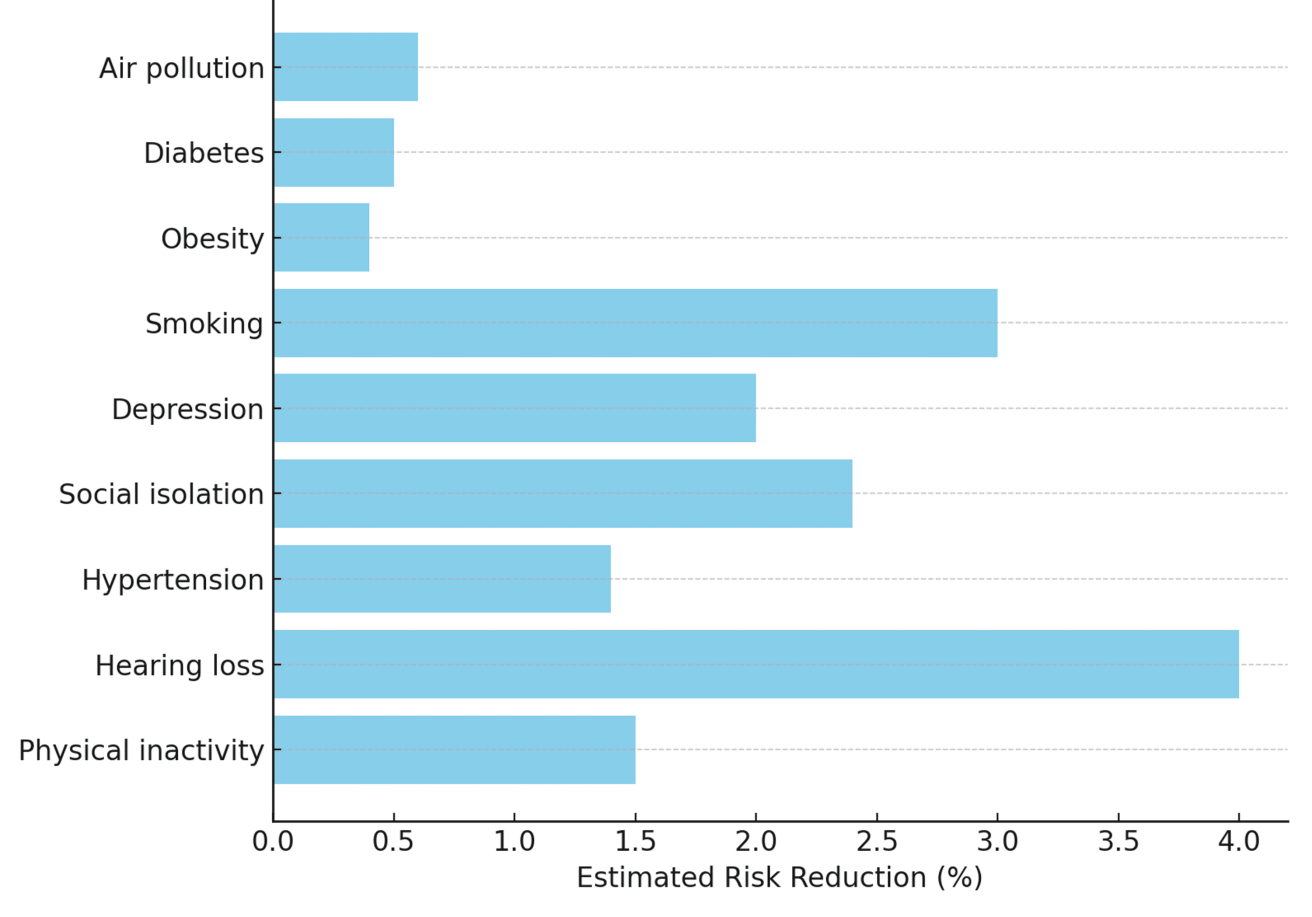
Estimated Individual Risk Reduction Contributions by Selected Modifiable Factors, Based on Modeled Relative Risk Reduction Multiplied by Intervention Effectiveness

Stratification by individual risk factor profiles showed that interventions addressing physical inactivity, hearing loss, hypertension, and social isolation had the largest impact on delaying progression (Figure 2). These effects were amplified when multiple risk domains were addressed concurrently, illustrating the value of a multifactorial prevention strategy.

Dropout remained low and linear over time, averaging about 2% annually, suggesting that digital therapeutic engagement is feasible in this population. Overall, the simulation supports the hypothesis that combining behavioral and clinical risk factor modification with digital engagement tools can substantially shift long-term cognitive outcomes in high-risk populations.

**Figure 3:**
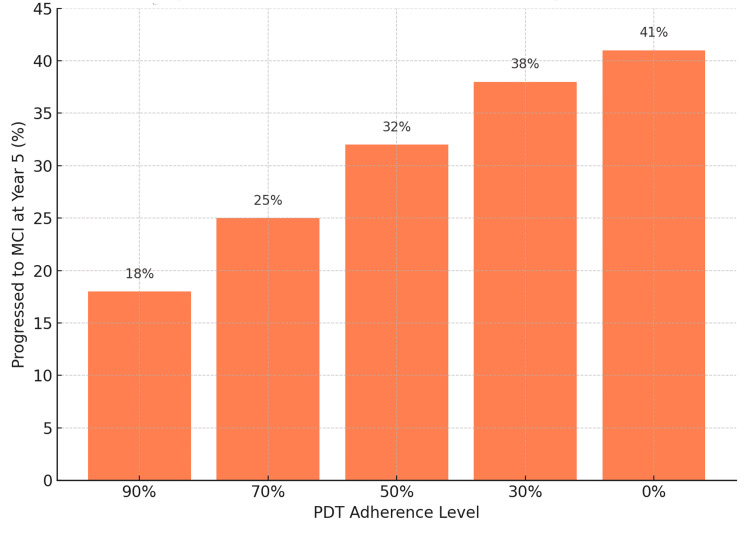
Sensitivity Analysis Showing the Percentage of Patients (n=10,000) Progressing to Mild Cognitive Impairment (MCI) by Year Five Based on Varying Levels of Adherence to the Prescription Digital Therapeutic (PDT)

The simulation demonstrated that full adherence to the digital therapeutic was associated with a substantial reduction in progression, particularly when combined with modification of prevalent risk factors such as physical inactivity, hearing loss, hypertension, and social isolation (Figure 3). Patients with multiple successfully modified risk factors exhibited the most favorable outcomes. The compounded benefit across modifiable domains showed a clear dose-response relationship, with greater risk reduction achieved in patients addressing two or more risk factors.

## Discussion

This simulation assumes a digital therapeutic capable of delivering a full-spectrum, multidomain intervention targeting modifiable dementia risk factors. By consolidating diverse interventions into a single app, the model reflects real-world trends toward integrated, app-based care that can support cognitive training, behavioral change, medical adherence, and social engagement in older adults. This unified approach is particularly relevant in the context of dementia prevention and progression, where multiple overlapping risks must be addressed simultaneously.

By leveraging the flexibility of a Monte Carlo simulation, we were able to test the additive and multiplicative effects of a multidimensional prevention strategy, incorporating adherence variability and modifiable vs non-modifiable risk factors. The results support the hypothesis that even modest improvements across several risk domains can yield significant protective effects at the population level. The inclusion of all modifiable dementia risk factors from the 2024 Lancet Commission update ensures a comprehensive and realistic modeling framework [4]. Model outputs were calibrated against observed real-world longitudinal progression rates in untreated SCD cohorts (10% annually), and the results from stratified subgroups align with protective effects found in prior longitudinal intervention studies [10,11,15].

Despite its strengths, the simulation has limitations. While risk factors were modeled as independent variables, this approach provides clarity in attributing individual contributions to overall risk reduction. Although in real-world settings factors such as obesity, hypertension, and diabetes often cluster together, isolating each variable avoids the complexity of subgroup analysis and supports transparent modeling of additive and multiplicative effects. Future iterations may incorporate conditional probability models to reflect risk factor clustering for even greater ecological validity. Similarly, the static dropout and adherence rates may not reflect the dynamics of real-world behavioral interventions. Future simulations may integrate conditional probability models and longitudinal adherence patterns to increase ecological validity.

Nonetheless, this model highlights the promise of scalable, smartphone-delivered prevention strategies that address the complexity of dementia risk in aging populations. The observed improvements in cognitive stability, even in a higher-risk cohort such as SCD, reinforce the need to translate such models into real-world pilot programs and pragmatic clinical trials.

## Conclusions

This study demonstrates the feasibility and impact of integrating PDTs with modifiable risk factor reduction to prevent cognitive decline in individuals with SCD. Using a comprehensive Monte Carlo simulation incorporating well-established dementia-related risk factors, we observed a marked reduction in progression from SCD to MCI compared to untreated populations. Future work should focus on building such an intervention, followed by rigorous prospective validation through clinical trials to confirm effectiveness, optimize adherence strategies, and assess long-term real-world impact. Additionally, incorporation of biomarker or imaging data may further enhance individual-level risk stratification and outcome prediction.
